# Myostatin Levels and the Risk of Myopenia and Rheumatoid Cachexia in Women with Rheumatoid Arthritis

**DOI:** 10.1155/2022/7258152

**Published:** 2022-05-10

**Authors:** Fabiola Gonzalez-Ponce, Jorge Ivan Gamez-Nava, Eli Efrain Gomez-Ramirez, Melissa Ramirez-Villafaña, Heriberto Jacobo-Cuevas, Norma Alejandra Rodriguez-Jimenez, Eva Maria Olivas-Flores, Yussef Esparza-Guerrero, Alejandro Martelli-García, Aline Priscilla Santiago-Garcia, Cesar Arturo Nava-Valdivia, Alejandra Martinez-Hernandez, Sergio Antonio Gonzalez-Vazquez, Alfredo Celis, Carlos Enrique Cabrera-Pivaral, Sylvia Totsuka-Sutto, Ernesto German Cardona-Muñoz, Laura Gonzalez-Lopez

**Affiliations:** ^1^Programa de Doctorado en Farmacología, Centro Universitario de Ciencias de la Salud (CUCS), Universidad de Guadalajara, 44340 Guadalajara, Jalisco, Mexico; ^2^Instituto de Terapeutica Experimental y Clínica, CUCS, Universidad de Guadalajara, 44340 Guadalajara, Jalisco, Mexico; ^3^Programa de Doctorado en Salud Publica, CUCS, Universidad de Guadalajara, 44340 Guadalajara, Jalisco, Mexico; ^4^Departamento de Microbiologia y Patología, CUCS, Universidad de Guadalajara, 44340 Guadalajara, Jalisco, Mexico; ^5^División de Disciplinas para el Desarrollo, Promoción y Preservación de la Salud, Departamento de Salud Pública, CUCS, Universidad de Guadalajara, 44340 Guadalajara, Jalisco, Mexico

## Abstract

**Background:**

Myostatin is a regulator of muscle size. To date, there have been no published studies focusing on the relation between myostin levels and myopenia in rheumatoid arthritis (RA).

**Objective:**

Evaluate the value of serum myostatin as a biomarker of cachexia and low skeletal muscle mass (LSMM) in RA patients, along with whether high serum myostatin is associated to these conditions after adjusting for potential confounders.

**Methods:**

This cross-sectional study included 161 female RA patients and 72 female controls. In the RA group, we assessed several potential risk factors for LSMM and rheumatoid cachexia. Dual-energy X-ray absorptiometry was used to quantify the skeletal muscle mass index (SMMI) (considering LSMM ≤ 5.5 kg/m^2^) and the presence of rheumatoid cachexia (a fat-free mass index ≤ 10 percentile and fat mass index ≥ 25 percentile of the reference population). Serum myostatin concentrations were determined by ELISA. To identify a cut-off for high serum myostatin levels, we performed ROC curve analysis. Multivariable logistic regression analysis was used to identify the risk factors for LSMM and rheumatoid cachexia. The risk was expressed as odds ratios (ORs) and their 95% confidence intervals (95% CIs).

**Results:**

Compared to the controls, the RA group had a higher proportion of LSMM and exhibited high serum myostatin levels (*p* < 0.001). ROC curve analysis showed that a myostatin level ≥ 17 ng/mL was the most efficient cut-off for identifying rheumatoid cachexia (sensitivity: 53%, specificity: 71%) and LSMM (sensitivity: 43%, specificity: 77%). In the multivariable logistic regression, RA with high myostatin levels (≥17 ng/mL) was found to increase the risk of cachexia (OR = 2.79, 95% CI: 1.24-6.29; *p* = 0.01) and LSMM (OR = 3.04, 95% CI: 1.17-7.89; *p* = 0.02).

**Conclusions:**

High serum myostatin levels increase the risk of LSMM and rheumatoid cachexia. We propose that high myostatin levels are useful biomarkers for the identification of patients in risk of rheumatoid cachexia and myopenia.

## 1. Introduction

Rheumatoid arthritis (RA) is a chronic inflammatory rheumatic disease clinically characterized by synovial joint inflammation, high levels of proinflammatory cytokines, autoantibodies (rheumatoid factor, anticyclic citrullinated peptide antibodies) and a chronic inflammatory response. This autoimmune disease causes pannus formation, leading to subchondral bone and joint cartilage erosions and diverse sequelae [1]. In addition to articular inflammatory characteristics, patients with RA frequently experience a decrease in skeletal muscle mass at a frequency ranging from 20% to 43% [2, 3]. Furthermore, a considerable proportion of patients with RA can develop rheumatoid cachexia, which is defined as muscle wasting with or without an increase in fat mass index (FMI) usually accompanied by a stable weight [4]. Rheumatoid cachexia is frequently observed in these patients, and a meta-analysis reported that its prevalence ranged from 15% to 32% [5]. Cachexia has been associated with the development of osteoporosis, an inadequate response to infection, deteriorating physical capacity, fatigue, and metabolic and cardiovascular morbidities. Although the pathogenesis of rheumatoid cachexia is multifactorial, some recent experimental studies have found that several myokines can influence the integrity of myocytes and self-regulation of muscle function [6]. Myokines are cytokines produced by myocytes that possess autocrine and para/endocrine functions; they regulate the metabolism of muscles and participate in the regulation of several functions in adipose tissues, the liver, and the brain [7]. One of the main myokines is myostatin, a protein that belongs to the transforming growth factor-*β* family, and it has been suggested to negatively regulate muscle growth [8]. Myostatin is expressed in several tissues, primarily in skeletal muscle and secondarily in cardiac muscle and adipose tissue [9]. Several investigations performed in experimental models have demonstrated that elevated myostatin concentrations inhibit fibroblast proliferation, induce atrophy in muscle tissues by increasing ubiquitin-proteasomal activity, and decrease the activity of the insulin-like growth factor-serine/threonine kinase (IGF-AKT) signalling pathway [9].

To date, there is growing interest from physicians and researchers regarding the effects of myostatin on skeletal muscle in patients with chronic diseases. Regarding RA, some works have investigated the relationship of myostatin with some relevant clinical variables [10–12]. Recently, we identified a relationship between high serum concentrations of myostatin and an increase in joint inflammation as well as a decrease in skeletal muscle mass in patients with RA [10]. However, that study had an insufficient sample size to evaluate whether a cut-off value for high myostatin levels could help to identify myopenia or to demonstrate an increase in the risk of rheumatoid cachexia [10]. To date, the relevance of elevated levels of this myokine in the myopenia observed in RA patients has not been proven, and some authors have reported conflicting results, making it difficult to determine the clinical value of serum myostatin; for example, Wada et al. observed that lower myostatin levels were associated with myopenia in patients with RA [11], while in an abstract published by Silva et al., there was no correlation found between myostatin concentrations and the quantity of appendicular lean mass or the fat mass indices [12]. However, the results of both studies contradict the experimental evidence regarding the effect of myostatin on regulating the growth of skeletal myocytes decreasing the number and size of muscle fibers [8]. These contradictory findings make it necessary to conduct additional studies to assess the relationship between myostatin and rheumatoid cachexia and low skeletal muscle mass controlling by confounders. Therefore, the objective of our study was to evaluate the value of high serum myostatin levels as a biomarker of rheumatoid cachexia and myopenia in women with RA and to assess whether the elevated serum levels of this myokine constitute a relevant risk factor for rheumatoid cachexia independent of other factors.

## 2. Patients and Methods

### 2.1. *Design*: A Cross-Sectional Study

### 2.2. Study Population

We included 233 Mexican Mestizo women from western Mexico: 161 women had RA, and 72 women without inflammatory rheumatic diseases were included as controls. All patients were recruited from an outpatient research department of a university centre (Universidad de Guadalajara). (Instituto de Terapeutica Experimental y Clinica, Centro Universitario de Ciencias de La Salud, University of Guadalajara) in Guadalajara city, Mexico. This study was performed by researchers of the Group for the Assessment of Prognosis Biomarkers in Autoimmune Disorders. The characteristics and members of this group had been published elsewhere [13]. This is a multidisciplinary group of researches estblished for the assessment of diagnostic tests, biomarkers, and prognosis and the treatment of chronic diseases. At this centre, a cohort of RA patients has been ongoing since 2010, recruiting persons primarily from the Mexican Mestizo population in the western part of Mexico (mostly from Guadalajara city, the second largest city of Mexico in population). Controls were Mexican Mestizo individuals from the same geographical area who were assessed at the same university centre for the prevention, diagnosis, or treatment of chronic diseases, mainly including persons of the community interested in being assessed for the prevention or early diagnosis of overweight/obesity, hypertension, endocrine diseases, chronic renal diseases, osteoarthritis, or osteoporosis, among others. Patients and controls were invited to participate from March 2020 to March 2021. All RA patients and controls signed a voluntary consent form prior to inclusion in the study. For RA patients, the inclusion criteria were patients aged ≥18 years who met the 1988 American College of Rheumatology criteria for RA [14]. For the control group, women of a similar age as those in the RA group were included. For both RA patients and controls, we excluded individuals with diabetes mellitus, chronic renal failure (serum creatinine >1.5 mg/dL), transaminase levels > 2-fold higher than the normal laboratory values, cancer, thyroid diseases, active infection, pregnancy, or lactation. Persons with hypertension were allowed to participate if the disease was considered controlled by their physicians.

The study was approved by the Ethics Committee of the Institute of Experimental and Clinical Therapeutics (INTEC) at the University Center of Health Sciences (CUCS), University of Guadalajara (approval code CEI/482/2019). This research protocol adhered to the tenets outlined in the Declaration of Helsinki given in Fortaleza, Brazil, 2013. All the persons included in the study signed a voluntary consent form prior to participating.

### 2.3. Clinical Evaluation of the RA Patients and Controls

We administered a structured questionnaire to assess the sociodemographic characteristics, lifestyle habits (sedentarism), menopausal status and duration since menopause, and hypertension. In all the participants, weight, height, and body mass index (BMI) (estimated with the Quetelet formula) were assessed [15]. We classified the participants as sedentary if they did not report performing physical exercise for a minimum of 150 to 300 minutes per week (of moderate intensity or, alternatively, 75 to 150 minutes per week of vigorous intensity) [16]. Menopause was considered the cessation of ovarian function in women who reported that their last menstruation was at least one year ago [17], and we excluded women who underwent oophorectomy for any reason.

### 2.4. Specific Assessment for RA Patients

RA patients were assessed with a structured questionnaire to identify the disease duration, history of medical treatment, and functional class of the patient. We evaluated the functional class according to the criteria validated by the American College of Rheumatology for global functional status in RA [18]. According to these criteria, stage I was defined as normal function, and stages II to IV were defined as a patient with a deteriorated physical function, with II being defined as limited avocational activities (recreational and/or leisure) and a handicap or limited motion at one or more joints, III being defined as limited vocational (work, school, and homemaking) and avocational activities but the ability to perform usual self-care activities, and IV being defined as a limited ability to perform usual self-care, being bedridden, or being dependent on a wheelchair as consequence of RA [18]. Disease activity was determined by the Disease Activity Score for 28 joints (DAS28-ESR). This index assesses the count of 28 swollen joints and 28 tender joints, and the subjective global assessment of the patient is based on a visual analogue scale of disease severity ranging from 0 to 100 mm and including the erythrocyte sedimentation rate (ESR) or C-reactive protein (CRP) [19]. For this study, we used the ESR (erythrocyte sedimentation rate) to compute the DAS28-ESR score (DAS28-ESR), and the patients were classified into two groups according to the severity of disease activity by this index [19]: (1) DAS28-ESR < 3.2, RA patients with low disease activity or remission, and (2) DAS28-ESR ≥ 3.2, RA patients having moderate or severe disease activity [19].

### 2.5. Dual X-Ray Absorptiometry Studies for the Determination of Low Skeletal Muscle Mass and Rheumatoid Cachexia

The measurement of body composition in all patients was carried out using dual-energy X-ray absorptiometry (DXA) with a densitometer iDXA GE LUNAR equipment (software encore version 16.0, Madison, WI, USA). The segmental lean mass of the arms and legs was obtained, and the skeletal muscle index (SMI) was computed ([arms + legs]/adjusted by height squared) [20]. According to the SMI, patients were classified as having a low skeletal muscle mass (SMI < 5.5 kg/m^2^) or normal skeletal muscle mass (SMI ≥ 5.5 kg/m^2^) [21]. In addition, we identified RA patients who had cachexia as those who had loss of skeletal muscle mass plus stable or increased fat mass following standardized criteria [22]. For the determination of rheumatoid cachexia in these patients, we determined the fat-free mass index (FFMI) and the fat mass index (FMI). According to Engvall's criteria, we classified individuals with rheumatoid cachexia according to whether they had an FFMI below the 10^th^ percentile plus FMI above the 25^th^ percentile or FMI normal based on the reference population [23]. In our population, the 10^th^ percentile of FFMI was 13.74 kg/m^2^, and the 25^th^ percentile of FMI was 11.27 kg/m^2^.

### 2.6. Serum Myostatin Determination

Serum samples obtained from RA patients and controls were deposited in the Eppendorf tubes coded for the anonymized measurement of myostatin. Therefore, to eliminate confirmation and observer biases, the researchers who measured myostatin serum levels were blinded to the clinical information including group or clinical characteristics. The code was revealed after the conclusion of the study for the statistical analysis. We quantified the serum myostatin levels by enzyme-linked immunosorbent assay (ELISA) with a commercial kit (Human Myostatin BioSource®). The measurement of myostatin using this kit has a coefficient of variation < 15%, specificity of 99%, and sensitivity of 1.0 ng/mL.

### 2.7. Determination of High Serum Myostatin Levels

To identify the cut-off for a high myostatin level, we conducted a percentile analysis of myostatin levels, and we chose a value for myostatin above the 75^th^ percentile as the cut-off. To validate this cut-off, we performed receiver operating characteristic (ROC) analysis using the presence of low muscle mass (IMME < 5.5 kg/m^2^) in RA patients as a classifier.

### 2.8. Statistical Analysis

According to the Kolmogorov–Smirnov test, the myostatin levels followed a nonparametric distribution; therefore, we used nonparametric statistics. Quantitative variables were expressed as medians (and ranges), and qualitative characteristics were expressed as frequencies (and %). For comparison of the quantitative variables between two groups, Mann–Whitney *U* tests were performed, and for qualitative variables, chi-square tests (or Fisher's exact tests if applicable) were performed. To identify the cut-off value for a high myostatin level in the RA group, we used the 75^th^ percentile value, corresponding to 17 ng/mL. To validate this cut-off value for classifying low muscle mass and rheumatoid cachexia, we examined the performance of these myostatin values using receiver operating characteristic (ROC) curves. The area under the curve when assessing those myostatin levels as a biomarker of rheumatoid cachexia was 0.538, and the area under the curve when assessing those myostatin levels as a biomarker of low muscle mass (myopenia) in RA was 0.605. The referred cut-off value was used to estimate the sensitivity, specificity, positive predictive value, and negative predictive value of high levels of myostatin in identifying low skeletal muscle mass or rheumatoid cachexia. We used a multivariable analysis to determine whether high myostatin levels are an important risk factor for myopenia or rheumatoid cachexia, two multivariable logistic regression models were developed. The first model was applied to identify the risk factors for low skeletal muscle mass (myopenia) in RA, and the second model was applied to identify the variables associated with the risk of rheumatoid cachexia. The covariates for these models were variables that were significant in the univariable analyses or variables that had biological plausibility for increasing the potential risk of developing the dependent variables. Adjusted odds ratios (ORs) and their 95% confidence intervals (95% CIs) were obtained as measures of risk in these models using the stepwise method. The significance level was set to *p* ≤ 0.05. All analyses were performed using the statistical software R version 4.1.1.

## 3. Results

In Table 1, we compare the characteristics of the RA patients and controls. A total of 161 women with RA and 72 women without rheumatic disease (control group) were included in this study. Among the 161 RA patients, 86 (53%) had low skeletal muscle mass, and 30 (19%) had rheumatoid cachexia. These groups were similar in terms of age, rate of employment, menopausal status, height, and hypertension. Weight and indices related to the quantity of fat (FFMI and FMI) were lower in RA patients than in controls (*p* < 0.001). The proportion of sedentary patients was higher in the RA group than in the control group (*p* = 0.05). Myostatin concentrations were also higher in the RA group than in the control group (*p* < 0.001). Regarding the cut-off value for high levels of myostatin, 34% of RA patients had levels ≥ 17 ng/mL compared with 4% of the controls (*p* < 0.001).

Regarding the treatments, almost all the RA patients had synthetic-DMARDs (*n* = 154, 96%), glucocorticoids were used by 96 patients (60%), and biologic DMARDs were used by only 17 patients (11%), of them 4 had rituximab, 1 abatacept, and 12 anti-TNF agents (10 had etanercept, and 2 adalimumab). Additionally, all the patients using biologic DMARDs had failure to combined therapy of synthetic-DMARDs. In Table 2, we compare the clinical characteristics of the RA patients with myopenia and the RA patients with normal skeletal muscle mass. RA patients with myopenia had a longer disease duration (*p* = 0.02), worse functional class (II-IV) (*p* = 0.005), more active disease state (DAS28-ESR ≥3.2, *p* = 0.003), a higher prevalence of rheumatoid cachexia (*p* = 0.001), and higher myostatin concentrations (*p* = 0.02). No significant differences were observed in terms of the frequency of the use of glucocorticoids (*p* = 0.92), specific synthetic disease-modifying drugs (DMARDs) (*p* = 0.25), or biologic DMARDs (*p* = 0.32) between these two groups (data not shown). The median DAS28-ESR in RA patients with a low muscle mass was 3.12 (range: 0.96-7.12), while that among RA patients with a normal muscle mass was 2.69 (range: 0.91-7.19, *p* = 0.002). Finally, RA patients with a low muscle mass had a higher frequency of myostatin levels ≥ 17 ng/mL than RA patients with normal muscle mass (43% vs. 23%, *p* = 0.006; data not shown).

In Table 3, we compare the clinical characteristics of RA patients with cachexia and RA patients without cachexia. Patients with rheumatoid cachexia had a lower SMI (*p* = 0.001) and a higher frequency of elevated myostatin levels (≥17 ng/mL) (*p* = 0.01). No differences were observed in the proportions of patients treated with glucocorticoids (*p* = 0.19), specific DMARDs (*p* = 0.35), or biologic DMARDs (*p* = 1.0) between these two groups (data not shown).

As shown in Table 4, we computed the utility values of high levels of myostatin (≥17 ng/mL) as a potential biomarker of low skeletal muscle mass and rheumatoid cachexia. For low muscle mass, this cut-off value had a sensitivity of 43%, a specificity of 77%, and an area under the curve (AUC) of 0.605 (95% CI: 0.517 to 0.692). For rheumatoid cachexia, this cut-off value had a sensitivity of 53%, a specificity of 71%, and an AUC of 0.538 (95% CI 0.424 to 0.652); other utility values are shown in Table 4.

Table 5 presents the results of multivariable logistic regression analysis to identify the risk factors for low muscle mass in RA adjusted by confounders. Using the stepwise method after adjusting for age, disease duration, and deteriorated functional class (II-IV), the risk factors for low muscle mass that remained in the model after adjusting for confounders were a moderate or high Disease Activity Score assessed by DAS28-ESR (*p* = 0.001) and high myostatin levels (≥17 ng/mL; *p* = 0.02). BMI was shown to be a protective factor for myopenia (*p* < 0.001). We performed a second model in the multivariable logistic regression analysis to identify the risk factors for rheumatoid cachexia in this model after adjusting for age, disease duration, deteriorated functional class (II-IV), BMI, and DAS28-ESR; high myostatin levels (≥17 ng/mL) remained the most relevant risk factor for rheumatoid cachexia (OR = 2.79, 95% CI: 1.24-6.29; *p* = 0.01) (data not shown).

## 4. Discussion

The most relevant findings of this study can be summarized as follows: (i) The prevalence of myopenia (LSMM) in RA patients was 53%. (ii) Rheumatoid cachexia was identified in 19% of these patients. (iii) Higher levels of myostatin were observed in RA patients compared to controls. (iv) Using a myostatin concentration ≥ 17 ng/mL for high levels of myostatin, we identified that both myopenia and rheumatoid cachexia were associated with levels higher than this cut-off. (v) Using the referred cut-off value of ≥17 ng/mL, the sensitivity and specificity of high myostatin concentrations for identifying myopenia were 43% and 77%, respectively, and for detecting rheumatoid cachexia they had a sensitivity of 53% and a specificity of 71%. (vi) Finally, after adjusting for confounders, high myostatin concentrations were identified as independent risk factors for low muscle mass (OR = 3.04, 95%CI = 1.14-8.10) and for rheumatoid cachexia (OR = 2.79, 95%CI = 1.17-7.89).

The prevalence of myopenia reported herein (53%) is similar to that reported by other authors in Turkey and Morocco [3, 24]; however, it is higher than that observed by other studies performed in the United States and France [25–27]. A recent meta-analysis performed by Dao et al. identified in 16 studies a pooled prevalence of low muscle mass/sarcopenia of 30.2% (95%CI = 24.2-36.2%) in 2,240 adults with RA [28]. Differences in ethnicity/race and epidemiological risk factors can influence the variability in the prevalence rates reported across studies.

The prevalence of rheumatoid cachexia has been reported by a few studies. In a meta-analysis by Santo et al. of 8 studies assessing rheumatoid cachexia (only 5 assessed using DXA), the prevalence of rheumatoid cachexia ranged from 1% to 53.9% among RA patients [5]. In our study, we found that 19% of our RA patients had rheumatoid cachexia. This figure is similar to that reported for other cohorts of RA patients in Latin America. Santo et al. found a prevalence from 13% to 30% in RA patients [29]. In this context, we previously reported a prevalence of rheumatoid cachexia of 14% in one study with a smaller sample of RA patients [10]. These differences in prevalence can be explained in part by the method of body composition assessment (DXA vs. bioelectrical impedance analysis or anthropometric measurements), the cut-offs used for diagnosis, the presence of comorbid diseases, and the disease characteristics of RA that can increase the risk of developing this condition (such as early vs. longer disease duration and a severe inflammatory status). However, the definition used for the diagnosis of rheumatoid cachexia has relevance in the differences of prevalence. For instance, Ångström et al. [30] reported the prevalence of cachexia in patients with early rheumatoid arthritis to be 24% using the Engvall criteria and 32% using the Elkan criteria [23, 31]. We choose for this study the Engvall criteria for the classification of patients with rheumatoid cachexia.

Regarding the high levels of myostatin, we identified that approximately one-third of these RA patients had high serum levels of myostatin compared to only 4% of the controls. Contrary to these results, Silva et al., in a published abstract, reported lower myostatin levels in RA patients than in controls [12]. However, the difference between the results of both studies cannot be explained yet because, to date, the results of Silva's study have not been published in their entirety. Myostatin can be increased by disease activity and other factors; therefore, Silva's study although has relevant information the lack of the description of other clinical and therapeutical variables limits the interpretation of their results.

Myostatin is a myokine member of the tumour growth factor *β* (TGF-*β*) family, which is also described as growth/differentiation factor 8 (GDF-8) [32]. In adulthood, myostatin is produced by myocytes and other tissues, including the heart, adipose tissue, liver, and mammary gland [33]. Myostatin exerts its effects through various signalling pathways [33–39]. For example, it has been shown that myostatin binds to activin receptor type II (ActRIIB), which acts through the Smad pathway, inducing the inhibitory protein Smad7 and establishing a negative feedback loop to suppress the growth of myocytes, producing a decrease in skeletal muscle mass [32–34]. On the other hand, the elevated expression of myostatin is also associated with an increase in the production of proinflammatory cytokines [37–40]. In vitro studies have demonstrated that myostatin promotes interleukin-1*β* expression by synovial fibroblasts through extracellular signal-regulated kinase-1 (ERK), c-Jun N-terminal kinase (JNK), and AP-1 signalling pathways that inhibit miR-21-5p [37]; likewise, myostatin induces tumour necrosis factor-*α* expression in synovial fibroblasts of RA through the phosphatidylinositol 3-kinase (PI3K)–Akt–AP-1 signalling pathway [38]. There is evidence regarding the proinflammatory effects of myostatin. In experimental studies of induced arthritis, myostatin regulates the recruitment of Th17 cells through increased levels of CCL20 on joint tissues, which subsequently induces an increase in IL-17 levels, contributing to the persistence of inflammation [39]. Other studies have demonstrated overexpression of myostatin in the synovial tissue of RA patients [37, 40]. However, an increase in the serum levels of this myokine has also been reported by us in RA, mainly in patients with moderate or severe disease activity [10].

According to our results, we have identified myostatin as a marker related to low muscle mass and rheumatoid cachexia. These associations between increased myostatin and myopenia have also been reported in men from healthy community-living older adults, supporting our findings [41, 42]. Only a few studies have been conducted in RA patients to assess this association [11, 12]. The first study was published as abstract by Silva et al. [12]. Silva et al. studied 122 females with RA and 30 subjects without rheumatic diseases and found no correlations between the myostatin levels and low muscle mass (assessed by the appendicular lean mass index measured by DXA) or between the severity of disease activity in RA (DAS28-ESR) and myostatin levels [12]. Wada et al., in a published abstract evaluating 96 RA patients of both sexes, contrary to our findings, identified a positive correlation between high skeletal muscle mass (measured by bioimpedance) and elevated serum myostatin levels, whereas myostatin levels correlated negatively with the severity of disease activity by DAS28-ESR [11]. Unfortunately, these two studies have not yet been fully reported, and therefore, we cannot interpret other possible factors related to the discrepancies in these results. These findings contrast with our results; we observed that an increase in myostatin is associated with low skeletal muscle mass. Several experimental models and findings in a nonrheumatic population indicate that higher concentrations of myostatin can be associated with muscle wasting [9, 41, 42], supporting the biologic plausibility of our findings. Additionally, we performed adjusted statistical analyses in order to exclude the effects of confounders in the potential relation between myostatin and low skeletal muscle mass or rheumatoid cachexia.

We also investigated the cut-off point to define high serum levels of myostatin as a potential biomarker for the identification of low muscle mass and rheumatoid cachexia. After establishing the cut-off value for myostatin levels at ≥17 ng/mL using ROC curves, we found an AUC of 0.605 for low muscle mass and an AUC of 0.538 for rheumatoid cachexia. The sensitivity and specificity obtained with this cut-off suggest that a high myostatin level can be used as a complementary biomarker of these conditions.

Finally, a high level of myostatin was identified as a risk factor for low muscle mass and rheumatoid cachexia. This increase in risk is based on the results of the multivariable analysis, the risk of myopenia (ORs) increased with moderate/severe disease activity, and high levels of myostatin (≥17 ng/mL). The increased risk of myopenia in patients with high disease activity in RA has been previously published by Ngeuleu et al. [24], and part of the mechanism linking myopenia with disease activity can be the increase of myostatin supporting our findings. The biological plausibility of these results is supported by evidence showing that disease activity and deteriorated functional class are related to the increase in proinflammatory cytokines such as interleukin 6, interleukin 1*β*, and TNF*α*, which can deteriorate muscle mass developing myopenia [43].

On the other hand, we identified that a high BMI was a protective factor that decreased the risk of myopenia; these results are supported by previous studies performed among RA patients [3, 24], although this finding was not observed by others [2]. A low BMI is associated with other relevant outcomes in RA; for instance, Fukuda et al. observed an association between a low BMI and poor quality of life [44]. Similarly, we identified in the multivariable analysis that high myostatin levels (≥17 ng/mL) increased the risk of rheumatoid cachexia, but no association was observed between the severity of the disease and cachexia. In a meta-analysis by Santo et al., they did not find any relation between cachexia and active disease or disease duration in RA [5]. Instead, two different studies performed by Santo et al. [29] and Ångström et al. [30] identified a possible relation between disease activity and changes in body composition parameters. This is an interesting result that deserves further evaluation in longitudinal studies.

Rheumatoid cachexia manifests as progressive muscle wasting with a stable or increased fat mass [23, 45]. Cachexia is a condition related to weight loss, where muscle tissue, adipose tissue, and bone tissue are affected, and it is mainly associated with the excessive production of cytokines, although other factors have also been described [46, 47]. Moreover, our findings show that a high myostatin level (≥17 ng/mL) is an independent risk factor for rheumatoid cachexia, although myostatin levels have not been previously evaluated in rheumatoid cachexia by other groups. Myopenia, cachexia and sarcopenia are associated to worse prognosis in RA including the development of articular damage, osteoporosis and osteoporotic fractures among others [48–50]. These results are consistent with those described in the literature on chronic wasting diseases and experimental studies [46, 51–53]. Myostatin plays a relevant role in the development of muscle atrophy by inducing cachexia in nonrheumatic patients and experimental studies [46]. In murine models, it has been observed that high doses of myostatin can decrease the myotube diameter in muscle [51]. Myostatin downregulates the expression of myogenic genes (MyoD) [52] and upregulates some of the genes involved in ubiquitination by mediating proteolysis and activating FoxO1 and atrogin-1, resulting in inactivation of the myogenic gene MyoD, favouring the development of a cachectic condition in experimental models [53].

Our study has several limitations that should be addressed by future studies. First, this work was designed to simultaneously evaluate myostatin concentrations and their relation to myopenia at a single point in time. This study design is useful to test the utility of a new potential marker, which was one of our objectives; however, information regarding the changes in myostatin levels over time could not be ascertained. Future longitudinal cohort studies should address this limitation to determine whether changes in myostatin levels are predictive of a clinically relevant decrease in skeletal muscle mass over the long term. A second limitation of our study is that some of the risk factors assessed in the present work can change over time; these factors might include modifications to the treatment regimen to include DMARDs or corticosteroid drugs, which could influence muscle mass. Again, prospective cohort studies are required to examine the effects of these factors on muscle mass. The third limitation is that we did not include men in our study, so the results reported in this study can only be extrapolated to the female population with RA. Finally, other works have identified a protective effect of anti-TNF agents or other biologic agents in rheumatoid cachexia [25, 54], however, in our study, only 17 patients were treated with biologic agents. This low frequency of treatments with biologics is frequent in Latin-American patients with RA by issues related to the elevated cost and low availability of these treatments in public hospitals without private insurance. Therefore, an important point to be assessed in future studies is the effect of these biologic agents on myostatin levels and the relation with rheumatoid cachexia.

The present study also has several strengths. This is the first study to propose myostatin level cut-off value to identify rheumatoid cachexia and low muscle mass and to investigate the contribution of high myostatin levels to the risk of developing these two conditions. Additionally, this study assessed the utility of the myostatin cut-off value in predicting the risk of myopenia and rheumatoid cachexia using multivariable models. Based on the results of this study, we consider that high levels of myostatin are related to an increase in the risk of rheumatoid cachexia and myopenia in RA, and we propose that the measurement of myostatin can be useful as an additional tool for clinicians aiming to identify patients at risk of these conditions. Myostatin, therefore, can be a therapeutic target in rheumatoid cachexia or severe myopenia, and the high levels of this myokine can identify the potential patients to consider for treatment with myostatin inhibitors. However, future studies are needed to validate the results of the present study, and prospective longitudinal studies should be performed to identify whether the baseline myostatin level could be utilized to predict differences in relevant outcomes of patients with regard to cachexia and myopenia.

## 5. Conclusions

We identified that a high myostatin level is a risk factor for low muscle mass and rheumatoid cachexia, and this high risk remains after controlling for confounders. On the other hand, a high myostatin level had a sensitivity of 53% and specificity of 71% for predicting rheumatoid cachexia. Therefore, a myostatin level ≥ 17 ng/mL can be considered a marker of low muscle mass and rheumatoid cachexia in RA patients, which can help physicians make related medical decisions and encourage future research.

## Figures and Tables

**Table 1:** comparison between selected characteristics of patients with RA vs controls.

	RA *n* = 161	Controls *n* = 72	*p* value
Age, median (range)	58 (18-89)	59 (36-73)	0.55
Scholarship ≤ high school	117 (73)	54 (75)	0.71
Employee, *n* (%)	44 (70)	19 (30)	0.88
Menopause, *n* (%)	132 (82)	60 (83)	0.80
Sedentary, *n* (%)	105 (65)	37 (51)	0.05
Weight (kg), median (range)	63.0 (39.5-96.0)	69.6 (52.8-96.6)	<0.001
Height (cm), median (range)	155 (137-170)	155 (142-171)	0.50
BMI (kg/m^2^), median (range)	26.3 (16.5-39.5)	28.3 (20.8-42.1)	<0.001
Arterial hypertension, *n* (%)	55 (70)	24 (33)	0.90
SMI (kg/m^2^)	5.44 (2.69-9.53)	6.58 (5.5-10.73)	<0.001
Low muscle mass (SMI < 5.5 kg/m^2^), *n* (%)	86 (53)	0 (0)	NA
FFMI (kg/m^2^)	13.7 (9.7-23.5)	15.7 (12.6-20.5)	<0.001
FMI (kg/m^2^)	12.2 (4.1-21.7)	13.2 (7.9-23.2)	0.004
Rheumatoid cachexia, *n* (%)	30 (19)	NA	NA
Myostatin (ng/mL)	11.89 (1.2-140)	7.9 (1.2-19.6)	<0.001
High myostatin (≥17 ng/mL)	54 (34)	3 (4)	<0.001

BMI: body mass index (kg/m^2^); SMI: skeletal muscle index; FFMI: fat-free mass index; FMI: fat mass index; NA: not applicable. Quantitative variables expressed in medians and ranges and compared by Mann–Whitney *U* tests; qualitative variables expressed in frequency and (%) and compared by chi-square tests.

**Table 2:** Comparison of clinical characteristics between RA patients with and without low muscle mass in the univariable analysis.

	RA+low muscle mass SMI < 5.5 kg/m^2^ *n* = 86	RA+normal muscle mass SMI ≥ 5.5 kg/m^2^ *n* = 75	*p* value
Age, median (range)	57.5 (18-80)	59.0 (24-89)	0.80
Menopause, *n* (%)	74 (86)	58 (77)	0.15
Sedentary, *n* (%)	57 (66)	48 (64)	0.76
BMI (kg/m^2^), median (range)	23.5 (16.5-36.7)	29.3 (21.4-39.5)	<0.001
Disease duration, median (range)	12 (1-40)	8 (1-35)	0.02
Deteriorated functional class (II-IV), *n* (%)	69 (80)	45 (60)	0.005
DAS28-ESR ≥ 3.2	41 (48)	19 (25)	0.003
Rheumatoid cachexia, *n* (%)	24 (28)	6 (8)	0.001
Myostatin (ng/mL), median (range)	13.54 (1.88-140)	10.02 (1.2-117)	0.02
High myostatin levels (≥17 ng/mL)	37 (43)	17 (23)	0.006

DAS28-ESR: Disease Activity Score (28 joints), Erythrocyte Sedimentation Rate. DAS28-ESR ≥ 3.2 indicates moderate or severe disease activity in RA patients. Quantitative variables expressed in medians and ranges and compared by Mann-Whiney *U* tests; qualitative variables expressed in frequency and % and compared by chi-square tests.

**Table 3:** Comparison of clinical characteristics between RA patients with and without cachexia in the univariable analysis.

	RA+cachexia *n* = 30	RA+noncachectic *n* = 131	*p* value
Age, median (range)	56 (42-80)	59 (18-89)	0.75
Menopause, *n* (%)	28 (93)	104 (80)	0.07
Sedentary, *n* (%)	20 (67)	85 (65)	0.85
BMI (kg/m^2^), median (range)	26.6 (23.1-29.4)	25.8 (16.5-39.5)	0.59
Disease duration, median (range)	13 (1-35)	10 (1-40)	0.75
Deteriorated functional class (II-IV), *n* (%)	23 (77)	91 (70)	0.43
DAS 28, median (range)	3.13 (1.27-5.05)	2.93 (0.91-7.19)	0.29
DAS28-ESR ≥ 3.2	14 (47)	46 (35)	0.24
SMI (kg/m^2^), median (range)	5.15 (4.29-6.03)	5.65 (2.69-9.53)	0.001
Low muscle mass, SMI < 5.5 kg/m^2^, *n* (%)	24 (80)	62 (47)	0.001
Myostatin (ng/mL), median (range)	17.59 (1.4-67.3)	11.4 (1.2-140)	0.52
High myostatin levels (≥17 ng/mL)	16 (53)	38 (29)	0.01

DAS28-ESR: Disease Activity Score (28 joints), Erythrocyte Sedimentation Rate. DAS28-ESR ≥ 3.2 indicates moderate or severe disease activity in RA patients. SMI: Skeletal Muscle Index. Quantitative variables expressed in medians and ranges and compared by Mann–Whitney *U* tests; qualitative variables expressed in frequency and % and compared by chi-square tests.

**Table 4:** Utility values of myostatin levels ≥ 17 ng/mL, for a biomarker of low muscle mass and rheumatoid cachexia.

Utility values of the assay	Low muscle mass	Rheumatoid cachexia
Sensitivity, % (95% CI)	43 (32-54)	53 (34-72)
Specificity, % (95% CI)	77 (66-86)	71 (62-79)
Positive predictive value, % (95% CI)	69 (57-78)	30 (22-39)
Negative predictive value, % (95% CI)	54 (49-60)	87 (82-91)
LR+	1.89 (1.17-3.08)	1.83 (1.19-2.82)
LR-	0.74 (0.59-0.91)	0.65 (0.44-0.97)
Prevalence	53 (45-61)	19 (13-26)

LR+: positive likelihood ratio; LR-: negative likelihood ratio.

**Table 5:** Associated factors with low muscle mass in RA.

	Univariate Intro method			Multivariate Stepwise method		
	OR	95% CI	p-value	OR	95% CI	*p* value
Age	1.01	0.97-1.05	0.59	—	—	—
BMI	0.62	0.52-0.73	<0.001	0.61	0.52-0.72	<0.001
DAS28-ESR	1.86	1.20-2.89	0.006	2.10	1.38-3.21	0.001
Disease duration	1.02	0.97-1.08	0.35	—	—	—
Deteriorated functional class	2.19	0.79-6.06	0.13	—	—	—
High myostatin (≥17 ng/mL)	3.04	1.14-8.10	0.03	3.04	1.17-7.89	0.02

Multivariable logistic regression analysis. Dependent variable presence of low muscle mass in RA patients. OR: odds ratios; 95% CI: 95% confidence intervals. Crude ORs were obtained using the Enter method. Adjusted ORs were obtained using the Forward stepwise method. BMI: body mass index (kg/m^2^); DAS28-ESR: Disease Activity Score (28 joints). Deteriorated functional class (II-IV).

## Data Availability

The data used to support the findings of this study are available on request through the author for correspondence: Dr. Laura Gonzalez-Lopez: lauraacademicoudg@gmail.com.
